# Genetic Analysis of Cardiac Syncope-Related Genes in Korean Patients with Recurrent Neurally Mediated Syncope

**DOI:** 10.3390/jcdd9080265

**Published:** 2022-08-14

**Authors:** Sung Ho Lee, Jong Eun Park, Chang-Seok Ki, Seung-Jung Park, Young Keun On, Kyoung-Min Park, June Soo Kim

**Affiliations:** 1 Division of Cardiology, Department of Internal Medicine, Kangbuk Samsung Hospital, Sungkyunkwan University School of Medicine, Seoul 03181, Korea; 2 Department of Laboratory Medicine, Hanyang University Guri Hospital, Hanyang University College of Medicine, Guri 11923, Korea; 3 GC Genome, Yongin 16924, Korea; 4 Division of Cardiology, Department of Medicine, Heart Vascular Stroke Institute, Samsung Medical Center, Sungkyunkwan University School of Medicine, Seoul 06351, Korea

**Keywords:** neurally mediated syncope, exome sequencing, syncope-related genes, *TTN*, *MYH7*

## Abstract

Neurally mediated syncope (NMS) is a common clinical problem. The underlying genetic factors of NMS remain controversial. We hypothesized that cardiac syncope-related genes may contribute to NMS in patients with previous frequent syncopal episodes and/or a family history of syncope. A total of 54 consecutive patients diagnosed with NMS were prospectively enrolled between 2013 and 2016. Inclusion criteria were more than five syncopal episodes with a family history of syncope (*n* = 17) or more than five syncopal episodes with no family history of syncope (*n* = 37). Ninety-eight cardiac syncope-related genes (channelopathy: 43 genes, cardiomyopathy: 50 genes, primary pulmonary hypertension: 5 genes) were screened by exome sequencing. All identified variants were classified according to the standards and guidelines by the American College of Medical Genetics and Genomics and the Association for Molecular Pathology. Of the 54 patients, 17 patients (31.5%) had a family history of syncope. Two patients (3.7%) had pathogenic and likely pathogenic variants (PV/LPV) in cardiac syncope-related genes *TTN* and *MYH7*. We investigated genetic variation in patients with frequent NMS with a positive family history of syncope in Korea. PV/LPVs in genes related to cardiomyopathy were associated with recurrent NMS in Korean patients. Closer follow-up of these patients might be needed.

## 1. Introduction

Neurally mediated syncope (NMS) is a common clinical problem that occurs in about 25% of the general population [1,2]. The mechanism of NMS remains unclear, but some researchers have suggested a genetic cause based on some familial forms of NMS [3,4,5,6]. Previous studies reported a frequency in family history of NMS from 19% to 50% [4,7,8]. Twin and familial studies revealed that NMS shows an autosomal dominant pattern [6]. Based on such a strong family history of syncope, some authors suggested a genetic involvement in the pathophysiological cascade leading to syncope [7,9]. In Western studies, an association between the cause of NMS and various single-nucleotide polymorphisms (SNPs) in sympathetic activity-related genes was suggested [9,10,11,12,13,14,15,16,17,18]; however, the genetic factors associated with NMS have not been elucidated and the results thus far have been conflicting [7,19,20]. There are many difficulties in explaining the cause of NMS based only on SNPs. The causes of syncope are diverse; some syncope may be associated with cardiac structural disease or channelopathy and the consequent increased risk of death [21]. It is important to identify the cause of syncope to establish an effective therapy. In this study, we hypothesized that cardiac syncope-related genes may contribute to NMS in patients with previous frequent syncopal episodes and/or a family history of syncope.

## 2. Materials and Methods

### 2.1. Study Population

A total of 150 consecutive patients with recurrent NMS were prospectively enrolled between March 2013 and September 2016. The diagnosis of NMS was made based on the clinical history and confirmed by a head-up tilt test and negative cardiologic evaluation (12-lead electrocardiogram, 24-h Holter, treadmill test, echocardiogram). All patients had experienced at least one episode of syncope within the 12 months before enrollment. Patients were free from any structural heart disease, arrhythmia, diabetes mellitus, or neurologic disease and were not taking any medication affecting autonomic function. Height and body weight were measured by trained staff. Body mass index was calculated as the weight in kilograms divided by the height in meters squared. Systolic blood pressure, diastolic blood pressure and heart rate at rest were measured. Questionnaires were used to determine current medication, and underlying diseases including cardiovascular disease, the number of previous syncopal or pre-syncopal episodes, triggering factors, seizure-like activity during syncope, syncope-related physical injury, prodromal symptoms, and presence or absence of a family history of syncope. Patients also provided information on syncope in first-degree and second-degree relatives. Among the total patient group, patients with frequent NMS were included in this study if they suffered more than 5 previous syncopal episodes (*n* = 37) or if they suffered more than 5 syncopal episodes and had a family history of syncope (*n* = 17). The study was approved by the regional Ethical Committee of the Samsung Medical Center (IRB No. 2012-11-005) and followed the Declaration of Helsinki. Informed consent was obtained from the study participants.

### 2.2. Whole-Exome Sequencing

Genomic DNA was extracted from peripheral blood leukocytes using a Wizard Genomic DNA purification kit (Promega, Madison, WI, USA) according to the manufacturer’s instructions. SureSelect Human All Exon V5 (Agilent Technologies, Santa Clara, CA, USA) was used for library preparation; sequencing was performed using the Illumina HiSeq2500 platform (Illumina Inc., San Diego, CA, USA). Alignment of the sequence reads was performed against the Human Reference Genome build GRCh37 using BWA 0.7.12; duplicated reads were marked with Picard Tools 1.130; the local alignment, base quality recalibration and variant calling were performed with the Genome Analysis Tool Kit v3.4.0; and annotation and variant effect prediction were performed with SnpEff v4.1g.

### 2.3. Variant Filtering Steps and Interpretation of Candidate Variants

The called variants were filtered and prioritized using a four-step strategy to generate a short candidate variant list for experimental validation (see Supplementary File S1: Figure S1). We first removed variants below 10× coverage. Variants were then limited to those with a low population frequency. Before removing <10× variants, we checked against ClinVar (https://www.ncbi.nlm.nih.gov/clinvar/, accessed on 5 April 2019) and the Human Gene Mutation Database (HGMD, http://www.hgmd.org/, accessed on 29 June 2022). Any previously reported pathogenic or probable pathogenic variants, whether or not these variants were <10×, were not filtered out. The minor allele frequency (MAF) threshold was carefully chosen and variants less than 0.01 were identified in the Genome Aggregation Database (gnomAD) (http://gnomad.broadinstitute.org/, accessed on 29 June 2022) or the Korean Reference Genome Database (KRGDB) (http://152.99.75.168/KRGDB, accessed on 29 June 2022). The next step was to include the variants predicted to have a high impact on protein function, including missense, nonsense, frameshifts, in-frame insertions/deletions variants, or changes affecting the consensus splice site sequences. Finally, we performed a gene-specific analysis with an in silico gene panel composed of 98 cardiac syncope-related genes (channelopathy: 43 genes, cardiomyopathy: 50 genes, primary pulmonary hypertension: 5 genes) (Table 1). These genes were obtained by searching previous publications [22,23].

Candidate variants were classified according to the standards and guidelines of the American College of Medical Genetics and Genomics (ACMG) and the Association for Molecular Pathology (AMP) [24]. These guidelines recommend classifying variants into five categories: pathogenic variant (PV), likely pathogenic variant (LPV), variant of uncertain significance (VUS), likely benign variant, and benign variant based on the combination of many lines of weighted evidence. The MYH7 gene, with ClinGen’s inherited cardiomyopathy expert panel recommendation, was additionally reflected when interpreting the ACMG/AMP guidelines [25]. To assess the frequency of a variant in the control or general population, we used the gnomAD, which consists of 125,748 exomes and 4359 genomes, and the KRGDB, which consists of 1722 Korean genomes. A primary literature review was conducted using various sources cited in the HGMD professional version (release February 2018), ClinVar, and PubMed to determine the potential pathogenicity of all identified variants. A variety of in silico tools were used to assess the predicted impact of missense change, including SIFT (http://sift.jcvi.org, accessed on 29 June 2022) and Polyphen2 (http://genetics.bwh.harvard.edu/pph2, accessed on 29 June 2022).

## 3. Results

### 3.1. Patient Characteristics

Clinical characteristics are shown in Table 2. Among the 54 patients, 36 (66.7%) were female; the mean patient age was 41.3 ± 8.9 years. The median number of previous syncopal episodes was six (interquartile range, 5–10). A total of 17 patients (31.5%) had a family history of syncope. The most common relative with a family history of syncope was the patient’s mother (24.1%), followed by sister (7.4%), aunt (3.7%) and son (1.9%). There was no history of syncope in relatives on the paternal side (patient’s father or brother).

Major syncope-related injuries, such as tooth fracture, were observed in two patients (3.7%); minor injuries such as contusions or lacerations were reported in thirty-one patients (57.4%). Thirty-five (64.8%) patients were classified as vasodepressive type, eleven (20.4%) patients as mixed type, and seven (13.0) patients as cardioinhibitory type according to Vasovagal Syncope International Study classification [26].

### 3.2. Genetic Characteristics of Subjects

Of the fifty-four patients, two patients (3.9%) had PV/LPVs according to the 2015 ACMG/AMP guidelines (Table 3). PVs were identified in *MYH7* and LPVs in *TTN*. A total of 113 VUSs were also identified (see Supplementary File S1: Tables S1–S3).

In a 34-year-old woman (PT-001) who had a family history of syncope involving her mother and sister and experienced syncope 10 times, an LPV (c.27755_27756insTCTTCTTTGTATG; p.Thr9253Leufs*23) was identified in the *TTN* gene; this is a novel variant that was absent from the control database. This variant was classified as an LPV with PVS1 and PM2 evidence. *TTN* can cause dilated cardiomyopathy (DCM) and a heterozygous truncated variant is the most common genetic cause of familial DCM [27].

In a 44-year-old woman (PT-104) who had a family history of syncope involving her son and experienced syncope five times, a PV (c.2608C>T;p.Arg870Cys) was identified in the *MYH7* gene. This PV was observed in an East Asian control (MAF of 0.006% in the gnomAD East Asian) and previously reported in familial hypertrophic cardiomyopathy (HCM) [28]. This variant was classified as a PV with PS4, PM1, PM2, PM5, and PP3 evidence. The *MYH7* gene is one of the major causative genes of HCM. According to Woo et al., a family affected by p.Arg870Cys had three premature cardiac deaths and one patient requiring myectomy among five family members.

## 4. Discussion

We analyzed 98 cardiac syncope-related genes in 54 patients with frequent recurrent NMS. Two PV/LPVs related to cardiomyopathy were detected in the *TTN* and *MYH7* genes. A total of 113 VUSs were identified in the 54 patients.

Previous genetic studies on NMS focused on the genes involved in cardiovascular reflex and the autonomic nervous system, affecting heart rate and vasoconstriction [7,20]; however, conflicting results have been reported regarding the genetic factors involved in the autonomic system [16,18,20,29,30,31]. These diverse results may be due to different study designs and methodologies regarding genes, ethnic differences, and different statistical analyses and sample sizes. Before the era of whole-exome sequencing, studies on SNPs associated with NMS might have had many limitations. In genetic studies, the significance level is usually set much lower than statistical *p* value significance (<0.05) and the results often come out as irrelevant [32]. Because GWAS platforms focus on common SNPs, effect sizes are often small and difficult to identify and validate unless a very large number of subjects is examined. In addition, SNPs associated with traits are usually not functional but rather serve as markers for loci that harbor truly functional variants.

Interestingly, two patients (3.9%) had PV/LPVs in the *TTN* and *MYH7* genes. *TTN* truncating variants have been suggested to cause 25% of DCM [27], and the *MYH7* gene, related to sarcomeres, may be present with a spectrum of phenotypical cardiomyopathies [HCM, DCM]) [33,34]. Although the patients in question had no clinical manifestations of cardiomyopathy, it would be considered carefully to extend surveillance of cardiomyopathy with electrocardiography or cardiac imaging to detect the development of phenotypically expressed cardiomyopathy [35]. However, there was also no family history of cardiomyopathy among their relatives.

Syncope might be the first manifestation of HCM in asymptomatic patients in the absence of LV hypertrophy on an echocardiogram [36]. Approximately 20% of HCM patients show a normal or near-normal LV mass on cardiac magnetic resonance imaging as a result of localized hypertrophy [37], and early stage HCM might not be diagnosed on conventional examinations. Thus, cardiomyopathy related syncope may be regarded as benign NMS because there is no evidence of structural and electrical abnormalities on conventional diagnostic examinations, or it might not be distinguished if there are combined overlapping NMS clinical features. Genetic testing could be an option for detecting early-stage cardiomyopathy. Follow-up of these genetic variant-related cardiomyopathy syncope patients over regular intervals with electrocardiography or imaging modalities such as echocardiography is critical to determine whether a cardiomyopathy phenotype will develop. Therefore, prolonged careful observation of these patients might be needed.

Our study has several limitations. First, the clear genetic causes of NMS are still unknown. Therefore, in this study, we had limitations in analyzing genes that were associated with NMS. NMS is also affected by environmental factors. Thus, gene-environment interactions could affect the predisposition to NMS. Second, a number of cardiomyopathy genes were found in a few patients, and cardiomyopathy-related genes themselves might not have clinical significance without the phenotype. Third, the study population sample size was relatively small. Further studies with more patients are needed. Lastly, family history data for syncope were self-reported by the patients. It is possible these patients may not be aware of every family member who suffered syncope and the number of affected family members may thus be underestimated. However, the unique point of this study is that we analyzed cardiac syncope-related genes among NMS patients with previous syncopal episodes and/or a family history of syncope and we found identified pathogenic variants for cardiomyopathy in these recurrent NMS patients.

In conclusion, we investigated genetic variation in patients with frequent NMS with a positive family history of syncope in Korea. PV/LPVs in genes related to cardiomyopathy were associated with recurrent NMS in Korean patients. Whole-exome sequencing provides information to facilitate genetic diagnosis and personalized treatment.

## Figures and Tables

**Table 1:** List of cardiac syncope-related genes.

Disease-Related Genes	Genes
Channelopathy genes (*n* = 43)	*KCNQ1, KCNH2, SCN5A, ANK2, KCNE1, KCNE2, KCNJ2, CACNA1C, CAV3, SCN4B, AKAP9, SNTA1, KCNJ5, CALM1, CALM2, CALM3, CACNB2, CACNA2D1, GPD1L, SCN1B, KCNE3, SCN3B, HCN4, KCND3, ABCC9, FGF12, KCND2, KCNE5, KCNJ8, RANGRF, SCN10A, SEMA3A, SLMAP, TRPM4, RYR2, CASQ2, TRDN, PRKAG2, NPPA, KCNA5, GJA5, NUP155, CDH2*
Cardiomyopathy genes (*n* = 50)	*PKP2, TGFB3, TMEM43, DSP, DSG2, DSC2, JUP, CTNNA3, DES, LMNA, PLN, TTN, TECRL, CALR3, ACTN2, LDB3, MYPN, FLNC, MYH7, MYLK2, MYL2, ACTC1, CSRP3, TNNC1, MYH6, VCL, MYOZ2, JPH2, TNNT2, NEXN, TPM1, MYBPC3, TNNI3, MYL3, PRDM16, PSEN2, RAF1, SGCD, LAMA4, EYA4, GATAD1, FKTN, RBM20, BAG3, CRYAB, PSEN1, DMD, ANKRD1, TMPO, TAZ*
Primary pulmonary hypertension genes (*n* = 5)	*BMPR2, SMAD9, CAV1, KCNK3, ACVRL1*

**Table 2:** Baseline characteristics of the study population.

Characteristic	Value (*n* = 54)
Age (years)	41.3 ± 8.9
Female (%)	36 (66.7)
Body weight (kg)	60.0 ± 10.9
Height (cm)	164.1 ± 7.7
Body mass index (kg/m^2^)	22.1 ± 3.0
Family history of syncope	17 (31.5)
Mother	13 (24.1)
Father	0
Brother	0
Sister	4 (7.4)
Aunt	2 (3.7)
Son	1 (1.9)
Number of syncopal episodes	6 (5–10)
Number of pre-syncopal episodes	2 (0–20)
Duration of syncope (years)	12.5 (8–22.2)
Diagnostic test	
12 lead electrocardiogram	
Normal sinus rhythm	50 (92.6%)
Sinus bradycardia	4 (7.4%)
Echocardiogram	
Ejection fraction (%)	61.8 ± 4.6
Head up tilt test	
Negative	1 (1.9)
Positive	53 (98.2)
Vasodepressor	35 (64.8)
Cardio-inhibition	7 (13.0)
Mixed	11 (20.4)
Syncope-related injury	30 (55.6)
Major injury (tooth fracture)	2 (3.7)
Minor injury	31 (57.4)
Contusion	17 (31.5)
Laceration	12 (22.2)
Seizure-like activity	6 (11.1)

Data are presented as mean ± standard deviation, number (%) or median (range).

**Table 3:** Genetic and clinical information of patients with recurrent syncope carrying likely pathogenic variants.

Patient ID	Gene	Nucleotide Change	Protein Change	Zyg	gnomAD East Asian	KRGDB	Polyphen-2	SIFT	ACMG Classification * (Evidences)	Number of Syncopal Episodes	Family History of Syncope
PT-001	*TTN*	c.27755_27756insTCTTCTTTGTATG	p.Thr9253Leufs*23	Het	N/A	N/A	N/A	N/A	LPV (PVS1, and PM2)	10	Mother and sister
PT-104	*MYH7*	c.2608C>T	p.(Arg870Cys)	Het	0.0001	0.0005	Probably damaging	Deleterious	PV (PS4, PM1, PM2, PM5, and PP3)	5	Son

Abbreviations: gnomAD, Genome Aggregation Database; Het, heterozygous; KRGDB, Korean Reference Genome Database; LPV, likely pathogenic variant; N/A, not applicable. * Identified variants were classified according to the standards and guidelines by the American College of Medical Genetics and Genomics and the Association for Molecular Pathology [24].

## Data Availability

All genomic variants identified in this study are available in Table 3 and Supplemental File Tables S1–S3. The original sequencing and clinical datasets generated during the current study are not publicly available due to patient confidentiality but are available from the corresponding author on reasonable request.

## Supplementary Materials

The following supporting information can be downloaded at: https://www.mdpi.com/article/10.3390/jcdd9080265/s1, Figure S1: Generation process of a candidate variant list using a four-step strategy; Table S1: List of variants of uncertain significance on in silico analysis of channelopathy genes; Table S2: List of variants of uncertain significance on in silico analysis of cardiomyopathy genes; Table S3: List of variants of uncertain significance on in silico analysis of primary pulmonary hypertension genes.
