# Supplementary material for: Genetic Analysis of Cardiac Syncope-Related Genes in Korean Patients with Recurrent Neurally Mediated Syncope
Source: J Cardiovasc Dev Dis. 2022 Aug 14;9(8):265. doi: 10.3390/jcdd9080265 (PMC9409957; doi:10.3390/jcdd9080265)
Supplement: Supplementary file 1 [file jcdd-09-00265-s001.zip › jcdd-1819276-supplementary.pdf]

Additional file 1

Figure S1: Generation process of a candidate variant list using a four-step strategy.

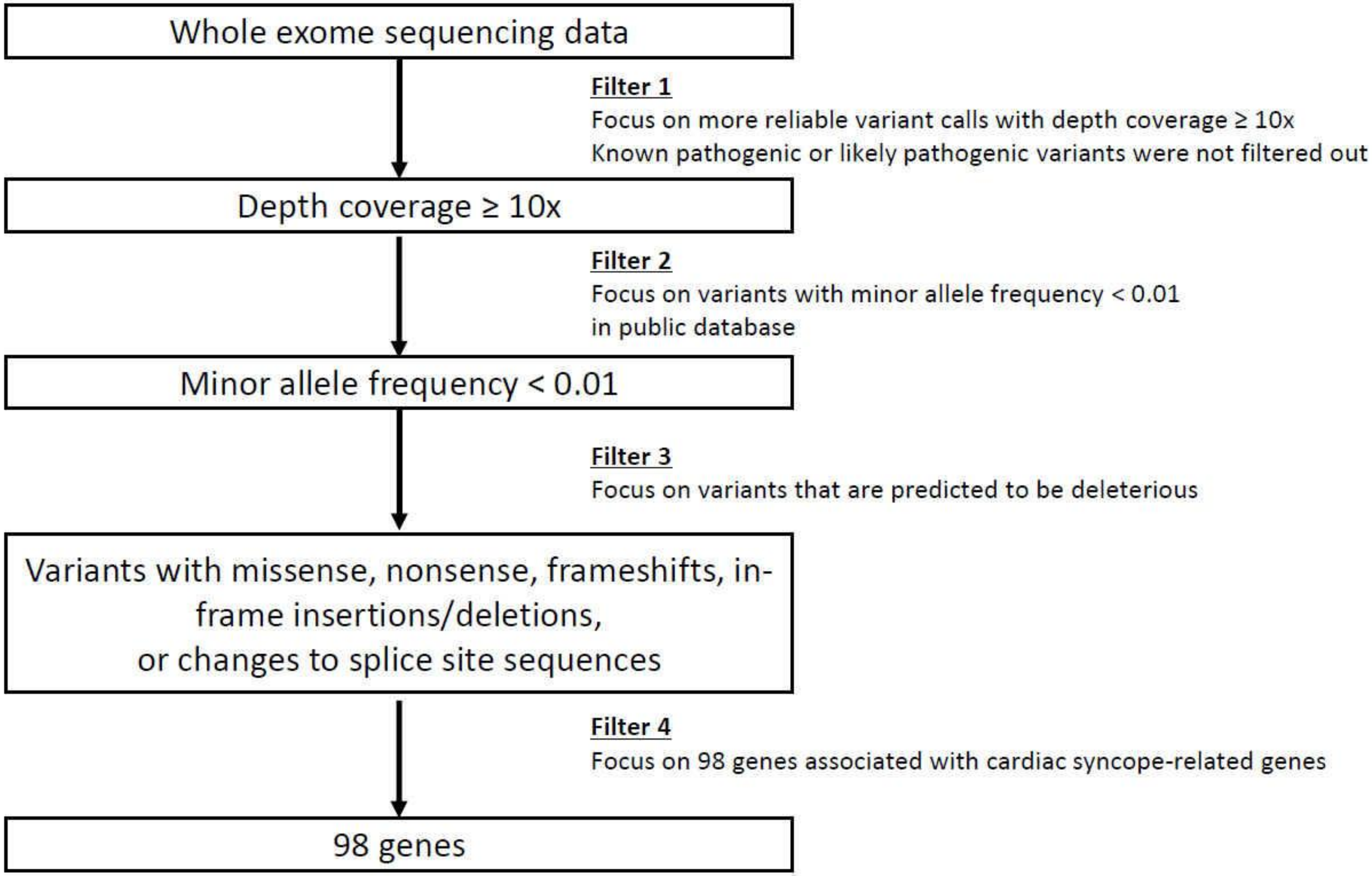

Table S1 List of variant of uncertain significance with in-silico analysis for channelopathy genes

Patient	Gene	Refseq	Nucleotide change	Amino acid change	Zyg	ACMG classification*	gnomAD_ALL	gnomAD_EA	SIFT	Polyphen2
PT-007	*ANK2*	NM_001148.4	c.10858T>A	p.(Trp3620Arg)	Het	VUS	2.85.E-05	4.07.E-04	Deleterious	Probably damaging
PT-008	*HCN4*	NM_005477.2	c.2845C>T	p.(Arg949Trp)	Het	VUS	1.42.E-04	1.14.E-03	Deleterious	Benign
PT-008	*TRPM4*	NM_017636.3	c.3304T>G	p.(Ser1102Ala)	Het	VUS	5.72.E-05	8.14.E-04	Tolerated	Benign
PT-010	*ANK2*	NM_001148.4	c.11425A>G	p.(Lys3809Glu)	Het	VUS	8.13.E-06	1.16.E-04	Deleterious	Benign
PT-010	*TRPM4*	NM_017636.3	c.3008C>T	p.(Pro1003Leu)	Het	VUS	2.85.E-05	2.90.E-04	Tolerated	Benign
PT-011	*SCN10A*	NM_006514.2	c.857C>T	p.(Thr286Ile)	Het	VUS	1.10.E-04	1.51.E-03	Deleterious	Benign
PT-011	*CACNA1C*	NM_000719.6	c.5747A>G	p.(Gln1916Arg)	Het	VUS	2.11.E-04	2.90.E-03	Deleterious	Benign
PT-014	*RYR2*	NM_001035.2	c.5046A>C	p.(Glu1682Asp)	Het	VUS	1.22.E-05	1.74.E-04	Deleterious	Benign
PT-014	*ANK2*	NM_001148.4	c.8015A>C	p.(Gln2672Pro)	Het	VUS	2.85.E-05	4.06.E-04	Deleterious	Probably damaging
PT-014	*TRPM4*	NM_017636.3	c.1408A>T	p.(Asn470Tyr)	Het	VUS	N/A	N/A	Tolerated	Benign
PT-019	*SCN10A*	NM_006514.2	c.3191C>T	p.(Thr1064Met)	Het	VUS	1.23.E-05	0.00.E+00	Deleterious	Benign
PT-019	*TRPM4*	NM_017636.3	c.2410G>A	p.(Asp804Asn)	Het	VUS	4.65.E-05	6.36.E-04	Deleterious	Probably damaging
PT-026	*AKAP9*	NM_005751.4	c.8189A>G	p.(Gln2730Arg)	Het	VUS	8.95.E-05	1.22.E-03	Tolerated	Benign
PT-027	*AKAP9*	NM_005751.4	c.11714T>C	p.(Met3905Thr)	Het	VUS	5.42.E-04	6.59.E-03	Tolerated	Benign
PT-036	*ANK2*	NM_001148.4	c.4A>G	p.(Met2Val)	Het	VUS	6.46.E-05	0.00.E+00	Deleterious	Benign
PT-039	*AKAP9*	NM_005751.4	c.3337A>T	p.(Met1113Leu)	Het	VUS	N/A	N/A	Tolerated	Benign
PT-049	*SCN10A*	NM_006514.2	c.1234G>A	p.(Glu412Lys)	Het	VUS	N/A	N/A	Tolerated	Probably damaging
PT-049	*AKAP9*	NM_005751.4	c.6184_6187delTTAG	p.(Leu2062Lysfs*3)	Het	VUS	N/A	N/A	N/A	N/A

PT-057	*SLMAP*	NM_007159.2	c.1174A>T	p.(Ile392Phe)	Het	VUS	3.73.E-05	0.00.E+00	Deleterious	Benign
PT-061	*AKAP9*	NM_005751.4	c.10406C>T	p.(Thr3469Met)	Het	VUS	3.14.E-04	3.71.E-03	Deleterious	Benign
PT-061	*ABCC9*	NM_005691.3	c.4604C>A	p.(Ala1535Asp)	Het	VUS	4.69.E-05	6.89.E-04	Tolerated	Benign
PT-061	*ABCC9*	NM_005691.3	c.4603G>A	p.(Ala1535Thr)	Het	VUS	7.22.E-05	7.43.E-04	Tolerated	Benign
PT-061	*TRPM4*	NM_017636.3	c.2410G>A	p.(Asp804Asn)	Het	VUS	4.65.E-05	6.36.E-04	Deleterious	Probably damaging
PT-066	*CACN/A1C*	NM_000719.6	c.1673C>T	p.(Thr558Met)	Het	VUS	1.27.E-04	1.00.E-03	Tolerated	Possibly damaging
PT-070	*AKAP9*	NM_005751.4	c.119G>A	p.(Arg40Lys)	Het	VUS	9.39.E-05	1.38.E-03	Tolerated	Benign
PT-083	*SCN4B*	NM_174934.3	c.231G>C	p.(Lys77Asn)	Het	VUS	4.06.E-06	5.80.E-05	Tolerated	Benign
PT-092	*SNTA1*	NM_003098.2	c.1123G>A	p.(Gly375Ser)	Het	VUS	2.86.E-05	2.33.E-04	Deleterious	Probably damaging
PT-104	*SEMA3A*	NM_006080.2	c.2200C>T	p.(Arg734Trp)	Het	VUS	2.44.E-05	1.74.E-04	Deleterious	Probably damaging
PT-105	*SLMAP*	NM_007159.2	c.1174A>T	p.(Ile392Phe)	Het	VUS	3.73.E-05	0.00.E+00	Deleterious	Benign
PT-108	*RYR2*	NM_001035.2	c.4244A>G	p.(Asp1415Gly)	Het	VUS	N/A	N/A	Tolerated	Benign
PT-113	*ANK2*	NM_001148.4	c.9689C>T	p.(Thr3230Met)	Het	VUS	3.67.E-05	3.48.E-04	Tolerated	Benign
PT-113	*NUP155*	NM_153485.2	c.3038A>G	p.(Asn1013Ser)	Het	VUS	1.63.E-05	2.32.E-04	Tolerated	Benign
PT-117	*SLMAP*	NM_007159.2	c.1174A>T	p.(Ile392Phe)	Het	VUS	3.73.E-05	0.00.E+00	Deleterious	Benign
PT-120	*KCND2*	NM_012281.2	c.906G>T	p.(Lys302Asn)	Het	VUS	6.52.E-05	5.90.E-05	Deleterious	Probably damaging
PT-121	*CASQ2*	NM_001232.3	c.362G>A	p.(Arg121His)	Het	VUS	2.44.E-05	0.00.E+00	Tolerated	Benign
PT-122	*SLMAP*	NM_007159.2	c.1174A>T	p.(Ile392Phe)	Het	VUS	3.73.E-05	0.00.E+00	Deleterious	Benign
PT-134	*CDH2*	NM_001792.3	c.1016G>A	p.(Arg339Gln)	Het	VUS	3.98.E-06	0.00.E+00	Deleterious	Probably damaging
PT-141	*SLMAP*	NM_007159.2	c.1174A>T	p.(Ile392Phe)	Het	VUS	3.73.E-05	0.00.E+00	Deleterious	Benign

gnomAD, Genome Aggregation Database; Het, heterozygous; N/A, not applicable; VUS, variant of uncertain significance; Zyg, zygosity.
*Identified variants were classified according to the standards and guidelines by the American College of Medical Genetics and Genomics and the Association for Molecular Pathology [1].

Table S2 List of variant of uncertain significance with in-silico analysis for cardiomyopathy genes

Patient	Gene	Refseq	Nucleotide change	Amino acid change	Zyg	ACMG classification*	gnomAD _ALL	gnomAD _EA	SIFT	Polyphen2
PT-006	*TTN*	NM_133378.4	c.20626C>T	p.(Pro6876Ser)	Het	VUS	4.89.E-05	6.97.E-04	Tolerated	Benign
PT-006	*RBM20*	NM_001134363.2	c.137C>T	p.(Pro46Leu)	Het	VUS	N/A	N/A	Tolerated	Benign
PT-006	*RBM20*	NM_001134363.2	c.226G>A	p.(Val76Ile)	Het	VUS	N/A	N/A	Tolerated	Benign
PT-007	*TTN*	NM_133378.4	c.72151C>T	p.(Arg24051Cys)	Het	VUS	3.66.E-05	1.16.E-04	Deleterious	Benign
PT-008	*ACTN2*	NM_001103.3	c.2554C>T	p.(Arg852Trp)	Het	VUS	4.07.E-06	0.00.E+00	Deleterious	Probably damaging
PT-008	*TTN*	NM_133378.4	c.3409G>A	p.(Gly1137Ser)	Het	VUS	2.44.E-05	1.16.E-04	Deleterious	Probably damaging
PT-008	*CALR3*	NM_145046.4	c.377C>T	p.(Ser126Leu)	Het	VUS	8.12.E-06	0.00.E+00	Deleterious	Probably damaging
PT-010	*DSP*	NM_004415.2	c.7021G>C	p.(Asp2341His)	Het	VUS	N/A	N/A	Deleterious	Probably damaging
PT-011	*TTN*	NM_133378.4	c.63833G>A	p.(Ser21278Asn)	Het	VUS	2.86.E-05	2.92.E-04	Deleterious	Benign
PT-011	*TTN*	NM_133378.4	c.18280G>A	p.(Asp6094Asn)	Het	VUS	2.05.E-05	2.94.E-04	Deleterious	Benign
PT-011	*TTN*	NM_133378.4	c.5813T>C	p.(Val1938Ala)	Het	VUS	3.25.E-05	2.90.E-04	Tolerated	Possibly damaging
PT-011	*PKP2*	NM_004572.3	c.131G>A	p.(Ser44Asn)	Het	VUS	1.00.E-05	1.30.E-04	Tolerated	Benign
PT-011	*TMPO*	NM_003276.2	c.682_684delTCC	p.(Ser228del)	Het	VUS	N/A	N/A	N/A	N/A
PT-012	*MYBPC3*	NM_000256.3	c.3316G>A	p.(Asp1106Asn)	Het	VUS	2.69.E-05	1.86.E-04	Deleterious	Probably damaging
PT-022	*TTN*	NM_133378.4	c.35586A>C	p.(Glu11862Asp)	Het	VUS	6.55.E-05	9.30.E-04	Deleterious	Probably damaging

PT-026	*TTN*	NM_133378.4	c.82655T>A	p.(Ile27552Asn)	Het	VUS	2.04.E-05	2.92.E-04	Deleterious	Possibly damaging
PT-026	*JUP*	NM_002230.2	c.427G>A	p.(Ala143Thr)	Het	VUS	9.51.E-05	2.33.E-04	Deleterious	Probably damaging
PT-027	*TTN*	NM_133378.4	c.82655T>A	p.(Ile27552Asn)	Het	VUS	2.04.E-05	2.92.E-04	Deleterious	Possibly damaging
PT-027	*TTN*	NM_133378.4	c.40274C>A	p.(Thr13425Asn)	Het	VUS	4.91.E-05	2.92.E-04	Tolerated	Benign
PT-032	*TTN*	NM_133378.4	c.86341C>T	p.(Arg28781Cys)	Het	VUS	8.96.E-05	1.16.E-04	Deleterious	Probably damaging
PT-032	*TTN*	NM_133378.4	c.17425A>C	p.(Thr5809Pro)	Het	VUS	6.97.E-05	1.17.E-04	Tolerated	Possibly damaging
PT-036	*TTN*	NM_133378.4	c.97702C>T	p.(Arg32568Trp)	Het	VUS	4.88.E-05	6.38.E-04	Deleterious	Probably damaging
PT-043	*TTN*	NM_133378.4	c.24124G>T	p.(Val8042Phe)	Het	VUS	2.90.E-05	3.49.E-04	Deleterious	Possibly damaging
PT-043	*TTN*	NM_133378.4	c.15583G>A	p.(Val5195Met)	Het	VUS	1.23.E-05	5.84.E-05	Tolerated	Possibly damaging
PT-049	*NEXN*	NM_144573.3	c.1688_1691dupGTAG	p.(Ser564Argfs*2)	Het	VUS	N/A	N/A	N/A	N/A
PT-051	*TTN*	NM_133378.4	c.12526G>A	p.(Asp4176Asn)	Het	VUS	4.08.E-06	5.85.E-05	Tolerated	Benign
PT-051	*FLNC*	NM_001458.4	c.5278G>A	p.(Gly1760Ser)	Het	VUS	1.85.E-04	6.72.E-04	Tolerated	Benign
PT-051	*FKTN*	NM_001079802.1	c.1182T>G	p.(Phe394Leu)	Het	VUS	4.06.E-06	5.80.E-05	Deleterious	Probably damaging
PT-056	*TTN*	NM_133378.4	c.48430C>T	p.(Pro16144Ser)	Het	VUS	1.23.E-05	1.76.E-04	Deleterious	Probably damaging
PT-056	*PKP2*	NM_004572.3	c.473G>A	p.(Arg158Lys)	Het	VUS	6.50.E-05	9.28.E-04	Tolerated	Benign

PT-056	*MYH6*	NM_002471.3	c.2306C>T	p.(Ala769Val)	Het	VUS	5.70.E-05	7.54.E-04	Deleterious	Possibly damaging
PT-057	*ACTN2*	NM_001103.3	c.1081A>G	p.(Met361Val)	Het	VUS	2.44.E-05	2.32.E-04	Tolerated	Benign
PT-064	*TTN*	NM_133378.4	c.90778A>G	p.(Ile30260Val)	Het	VUS	4.07.E-05	5.80.E-04	Tolerated	Benign
PT-064	*DSP*	NM_004415.2	c.4886G>T	p.(Ser1629Ile)	Het	VUS	5.32.E-05	7.56.E-04	Deleterious	Benign
PT-064	*MYH6*	NM_002471.3	c.3520C>T	p.(Gln1174*)	Het	VUS	1.35.E-05	1.19.E-04	N/A	N/A
PT-066	*TTN*	NM_133378.4	c.40036G>C	p.(Val13346Leu)	Het	VUS	5.74.E-05	8.09.E-04	Deleterious	Benign
PT-068	*TTN*	NM_133378.4	c.57212G>A	p.(Arg19071Gln)	Het	VUS	2.85.E-05	5.81.E-05	Deleterious	Probably damaging
PT-072	*TTN*	NM_133378.4	c.47705C>T	p.(Ala15902Val)	Het	VUS	4.26.E-06	6.09.E-05	Tolerated	Benign
PT-072	*DSP*	NM_004415.2	c.2540C>G	p.(Pro847Arg)	Het	VUS	3.66.E-05	5.22.E-04	Tolerated	Benign
PT-075	*FKTN*	NM_001079802.1	c.49A>C	p.(Ser17Arg)	Het	VUS	1.63.E-05	2.32.E-04	Deleterious	Probably damaging
PT-075	*MYBPC3*	NM_000256.3	c.2807C>T	p.(Thr936Met)	Het	VUS	4.08.E-05	0.00.E+00	Deleterious	Probably damaging
PT-077	*PRDM16*	NM_022114.3	c.43G>A	p.(Gly15Ser)	Het	VUS	2.89.E-05	1.75.E-04	Tolerated	Benign
PT-077	*TTN*	NM_133378.4	c.34406A>T	p.(Gln11469Leu)	Het	VUS	N/A	N/A	Deleterious	Probably damaging
PT-080	*PRDM16*	NM_022114.3	c.1726G>A	p.(Glu576Lys)	Het	VUS	1.47.E-04	9.86.E-04	Tolerated	Benign
PT-080	*TECRL*	NM_001010874.4	c.308G>A	p.(Arg103Gln)	Het	VUS	8.16.E-06	1.16.E-04	Deleterious	Probably damaging
PT-080	*MYBPC3*	NM_000256.3	c.3137C>T	p.(Thr1046Met)	Het	VUS	6.26.E-05	6.96.E-04	Tolerated	Benign
PT-083	*TTN*	NM_133378.4	c.26873C>T	p.(Pro8958Leu)	Het	VUS	1.25.E-05	1.79.E-04	Deleterious	Probably damaging
PT-085	*TTN*	NM_133378.4	c.28055T>C	p.(Val9352Ala)	Het	VUS	2.44.E-05	2.32.E-04	Tolerated	Benign
PT-085	*MYH6*	NM_002471.3	c.2384G>A	p.(Arg795Gln)	Het	VUS	4.47.E-05	1.16.E-04	Tolerated	Probably

										damaging
PT-091	*TTN*	NM_133378.4	c.80702C>T	p.(Ala26901Val)	Het	VUS	9.38.E-05	5.84.E-04	Tolerated	Benign
PT-091	*TTN*	NM_133378.4	c.41663G>A	p.(Arg13888His)	Het	VUS	4.98.E-05	1.17.E-04	Deleterious	Possibly damaging
PT-092	*PRDM16*	NM_022114.3	c.1453T>C	p.(Phe485Leu)	Het	VUS	4.08.E-06	5.80.E-05	Tolerated	Possibly damaging
PT-092	*FLNC*	NM_001458.4	c.1310G>T	p.(Arg437Leu)	Het	VUS	1.22.E-05	1.74.E-04	Tolerated	Benign
PT-094	*TTN*	NM_133378.4	c.2581G>A	p.(Ala861Thr)	Het	VUS	N/A	N/A	Deleterious	Benign
PT-094	*GATAD1*	NM_021167.4	c.117_131delGGGCAGCGGGGGCGC	p.(Gly40_Ala44del)	Het	VUS	9.08.E-05	0.00.E+00	N/A	N/A
PT-094	*DMD*	NM_004006.2	c.981C>G	p.(Asp327Glu)	Het	VUS	N/A	N/A	Tolerated	Benign
PT-095	*TTN*	NM_133378.4	c.21913C>T	p.(Pro7305Ser)	Het	VUS	3.73.E-05	5.19.E-04	Deleterious	Probably damaging
PT-100	*TTN*	NM_133378.4	c.40036G>C	p.(Val13346Leu)	Het	VUS	5.74.E-05	8.09.E-04	Deleterious	Benign
PT-100	*MYBPC3*	NM_000256.3	c.3750C>G	p.(Ile1250Met)	Het	VUS	1.22.E-05	1.74.E-04	Deleterious	Benign
PT-104	*TTN*	NM_133378.4	c.160G>A	p.(Val54Met)	Het	VUS	4.06.E-05	5.81.E-04	Deleterious	Benign
PT-105	*TTN*	NM_133378.4	c.23102T>C	p.(Met7701Thr)	Het	VUS	N/A	N/A	Deleterious	Benign
PT-113	*TTN*	NM_133378.4	c.36285G>T	p.(Lys12095Asn)	Het	VUS	1.22.E-05	1.76.E-04	Deleterious	Benign
PT-117	*TTN*	NM_133378.4	c.23129C>T	p.(Pro7710Leu)	Het	VUS	4.17.E-06	5.96.E-05	Deleterious	Probably damaging
PT-117	*TTN*	NM_133378.4	c.19280C>G	p.(Ala6427Gly)	Het	VUS	4.08.E-06	5.80.E-05	Tolerated	Benign
PT-117	*TTN*	NM_133378.4	c.16832G>C	p.(Arg5611Thr)	Het	VUS	N/A	N/A	Tolerated	Benign
PT-117	*MYH6*	NM_002471.3	c.1885G>A	p.(Asp629Asn)	Het	VUS	8.18.E-05	8.70.E-04	Tolerated	Possibly damaging
PT-118	*LAMA4*	NM_002290.4	c.572G>A	p.(Gly191Glu)	Het	VUS	3.26.E-05	4.66.E-04	Deleterious	Probably damaging

PT-122	*TTN*	NM_133378.4	c.91447G>A	p.(Glu30483Lys)	Het	VUS	2.03.E-05	2.90.E-04	Tolerated	Benign
PT-122	*PSEN1*	NM_000021.3	c.367G>A	p.(Glu123Lys)	Het	VUS	8.12.E-06	5.80.E-05	Tolerated	Benign
PT-130	*LAMA4*	NM_002290.4	c.1852A>G	p.(Asn618Asp)	Het	VUS	3.25.E-05	4.64.E-04	Deleterious	Possibly damaging
PT-130	*MYH6*	NM_002471.3	c.404A>G	p.(Asn135Ser)	Het	VUS	1.62.E-05	5.80.E-05	Tolerated	Benign
PT-133	*TTN*	NM_133378.4	c.29432C>T	p.(Pro9811Leu)	Het	VUS	2.93.E-05	2.43.E-04	Deleterious	Benign
PT-134	*BAG3*	NM_004281.3	c.1015A>G	p.(Ile339Val)	Het	VUS	4.06.E-06	5.80.E-05	Deleterious	Probably damaging

gnomAD, Genome Aggregation Database; Het, heterozygous; N/A, not applicable; VUS, variant of uncertain significance; Zyg, zygosity.

*Identified variants were classified according to the standards and guidelines by the American College of Medical Genetics and Genomics and the Association for Molecular Pathology [1].

Table S3 List of variant of uncertain significance with in-silico analysis for primary pulmonary hypertension genes

Patient	Gene	Refseq	Nucleotide change	Amino acid change	Zyg	Interpretation	gnomAD_ALL	gnomAD_EA	SIFT	Polyphen2
PT-001	*BMPR2*	NM_001204.6	c.1430A>G	p.(Lys477Arg)	Het	VUS	N/A	N/A	Tolerated	Probably damaging
PT-006	*BMPR2*	NM_001204.6	c.276A>C	p.(Gln92His)	Het	VUS	5.69.E-05	7.54.E-04	Tolerated	Benign

gnomAD, Genome Aggregation Database; Het, heterozygous; N/A, not applicable; VUS, variant of uncertain significance; Zyg, zygosity.

*Identified variants were classified according to the standards and guidelines by the American College of Medical Genetics and Genomics and the Association for Molecular Pathology [1].

REFERENCE

1. Richards S, Aziz N, Bale S, Bick D, Das S, Gastier-Foster J, et al. Standards and guidelines for the interpretation of sequence variants: a joint consensus recommendation of the American College of Medical Genetics and Genomics and the Association for Molecular Pathology. Genet Med 2015;17:405-24.
